# Effects of a Home-Based Rehabilitation Exercise Program on Cardiorespiratory Performance in Community-Dwelling Adults Who Underwent Heart Surgery: Randomized Controlled Trial

**DOI:** 10.2196/68504

**Published:** 2025-03-28

**Authors:** Natsinee Sermsinsaithong, Kornanong Yuenyongchaiwat, Chusak Thanawattano, Chatchai Buekban, Chitima Kulchanarat, Sasipa Buranapuntalug, Khanistha Wattanananont, Opas Satdhabudha

**Affiliations:** 1 Department of Physical Therapy, Faculty of Allied Health Sciences, Thammasat University, 99 Moo 18, Paholyothin Road, Klong Luang, Pathum Thani, 12120, Thailand, 66 824521680; 2 Thammasat University Research Unit, Physical Therapy in Respiratory and Cardiovascular Systems, Thammasat University, Pathum Thani, Thailand; 3 Biomedical Electronics and Systems Research Team, Assistive Technology and Medical Devices Research Group, National Electronics and Computer Technology Center, Pathum Thani, Thailand; 4 Physical Therapy Center, Thammasat University Hospital, Pathum Thani, Thailand; 5 Cardiac Rehabilitation Unit, Excellence Center, Faculty of Medicine, Navamindradhiraj University, Bangkok, Thailand; 6 Department of Surgery, Faculty of Medicine, Thammasat University, Pathum Thani, Thailand

**Keywords:** virtual reality, cardiac surgery, cardiac rehabilitation, cardiopulmonary, aerobic exercise

## Abstract

**Background:**

Patients undergoing heart surgery demonstrate impaired cardiorespiratory performance. Phase II cardiac rehabilitation (CR) in people undergoing open heart surgery (OHS) aims to reduce the adverse physical effects of cardiovascular diseases. Virtual reality (VR) exercise is now used in CR.

**Objective:**

This study aimed to explore the effects of VR exercise on functional capacity, pulmonary function, and respiratory muscle strength in patients who underwent OHS and were in phase II CR.

**Methods:**

Forty-nine patients who underwent elective OHS and were in phase II CR were randomized into a VR group (N=24) and a control group (N=25). The VR group completed 8 weeks of a home-based VR exercise program, including chest trunk mobilization and aerobic circuit training for 30 minutes, whereas the control group received an exercise brochure and information regarding the benefits of exercise. Intention-to-treat analysis was conducted, and 2-way mixed ANOVA was performed to compare between- and within-group differences in functional capacity and respiratory performance.

**Results:**

After completing the 8-week program, the VR group showed significant improvement in functional capacity compared to the control group (66.29, SD 25.84 m; *P*=.01). Inspiratory muscle strength increased in both the VR and control groups compared to baseline (9.46, SD 2.85 and 9.64, SD 2.78 cm H_2_O, respectively). In addition, after the 8-week intervention, significant improvements were found in expiratory muscle strength (15.79, SD 4.65 cm H_2_O) and forced expiratory volume in 1 second as a percentage of predicted values (2.96%, SD 1.52%) in the VR group compared to the baseline session.

**Conclusions:**

The home-based VR exercise program significantly improved functional capacity but not respiratory muscle or pulmonary function.

## Introduction

In 2020 and 2021, over three-quarters of cardiovascular diseases (CVD) and related deaths occurred in low- and middle-income countries [1]. In high-income countries, the average prevalence of total heart surgery is 123.2 per 100,000 population annually. The average coronary artery bypass graft (CABG) and valve surgery volumes are 36.7 and 30.8 per 100,000 population per year, respectively [2]. High- to middle-income countries show a higher rate of all cardiac surgeries than low- to middle-income countries and low-income countries [2].

Cardiac rehabilitation (CR) is recommended for patients who have heart disease and/or undergo cardiac surgery during admission at the hospital or after discharge [3-5]. CR programs aim to reduce mortality and improve mobility and the quality of life [4]. A systematic review and meta-analysis showed that CR programs in hospital-based, community-based, or home-based settings reduced mortality and hospitalization rates and improved the quality of life in patients with coronary artery disease, angina, or ischemic heart disease and undergoing CABG or percutaneous coronary intervention [6]. A study showed that patients who underwent CR exhibited a reduction in total mortality of 13%-24% within 1-3 years after a cardiac event and reduced readmission of 31% a year later [7].

The roles of physical therapy are associated with CR, mainly to improve physical activity and prescribe exercise to patients with cardiac disorders or diseases. Although exercise is essential for patients with CVD or those who have undergone cardiac surgery, CR remains extensively underused because of issues with adherence to CR programs and low participation [8]. Center-based CR participation rates among eligible patients are still low, ranging from 10% to 30% globally, possibly because of issues with accessibility, competing obligations, poor socioeconomic status, cost, and availability [9]. Adherence to CR programs is affected by several factors, for example, female sex, older age, low socioeconomic status, and lack of suggestions from a physician [4]. Further, adherence rates of 36.7%‐84.6% and dropout rates of 12%‐56% were observed in patients who engaged in CR programs. A systematic review of 43 prospective cohort studies with 63,425 patients from 10 different countries reported factors related to nonparticipation or dropout from CR, including older age, female sex, low socioeconomic status, comorbid conditions, physical inactivity, poor functional capacity, low exercise capacity, and refusal to participate. Moreover, motivational factors can affect patients’ compliance with CR [10].

For several decades, virtual reality (VR) technology has been used in combination with home-based exercise CR as an alternative for outpatients with cardiac disorders or diseases to reduce barriers to CR participation (eg, long distance from CR facilities and motivation), make rehabilitation more accessible, and enable the effective and safe performance of rehabilitation exercises [9,11].

VR allows people to engage with scenarios in virtual environments. The effects of VR exercise have also been shown to be useful in CR in several studies; VR has shown efficacy in preventing the loss of pulmonary function and promoting physical ability after phase I CABG [12]. In patients with phase II CABG, a VR exercise protocol for an in-hospital CR program increased peak oxygen consumption, peak metabolic equivalents, and the anaerobic threshold, which represents a positive effect on exercise tolerance [13]. Furthermore, VR during an 8-week intervention program improved metabolic equivalents, 6-minute walk distance (6MWD), and quality of life and reduced depression in patients with ischemic heart disease [14]. Thus, using VR to exercise may be an alternative method of CR.

There are several barriers to CR referral after patients are discharged from the health care system, leading to low CR participation rates. Alternative models have been developed in response to the challenges associated with the delivery of center-based CR, such as cost and accessibility. Moreover, VR exercise has recently been implemented in Thailand, although its advantages in patients who have undergone heart surgery have not been assessed. Therefore, this study hypothesized that home-based exercise would affect cardiovascular endurance, pulmonary function, and respiratory muscle strength in patients undergoing open heart surgery (OHS) who are involved in phase II VR exercise. This study aimed to evaluate the effects of a home-based exercise program on cardiovascular endurance and respiratory performance in patients undergoing OHS.

## Methods

### Overview

Community-dwelling patients who underwent OHS and were in phase II CR (defined as ≥3 weeks after discharge from the hospital) were recruited in this study. G*Power (version 3.1.9.4; Heinrich-Heine-Universität Düsseldorf) was used to determine the sample size with an effect size of 0.25, a statistical power of 0.90, and an α of .05; therefore, 46 participants were enrolled. Ten percent of the total sample size was estimated to account for data dropout or technical errors. Thus, 50 participants were recruited for this randomized controlled trial. Participants who underwent heart surgery via median sternotomy and were within 3‐6 weeks post discharge from the hospital or in phase II CR were invited to participate in the study. The exclusion criteria were unstable angina or acute myocardial infarction within the previous 3 months. Moreover, participants with an uncontrollable resting systolic blood pressure of >180 mm Hg and/or diastolic blood pressure of >120 mm Hg [15], resting respiratory rate of >30 bpm, cognitive impairment (measured by the Montreal Cognitive Assessment), or musculoskeletal conditions that affected walking were excluded. Participants who were admitted to the hospital or had active respiratory disease (eg, COVID-19) during the intervention program were required to withdraw from the study.

Eligible participants were allocated to 2 groups using simple random sampling: 25 individuals in the VR group and 25 in the control group. The same physiotherapist performed the assessment, and the participants were blinded to the group allocation. Participants who underwent VR exercise were required to attach an optical heart rate (HR) sensor to the left side above their elbow, and their resting HR was displayed on a screen (ie, a television or computer). Moderate-intensity exercise (ie, 40%‐59% HR reserve [HRR]; the maximal HR was determined by subtracting the participants’ ages from 220) was decided for each individual [16]. The VR program (ie, intervention) involved moderate-intensity exercise at a prescribed 40%‐59% HRR or a rate of perceived exertion of 11‐13 of 20, 2‐3 times a week, over 8 weeks (Table 1). The control group received no rehabilitation program; however, an exercise brochure and information regarding the benefits of exercise were provided.

**Table 1.** Protocol for the home-based rehabilitation exercise in phase II cardiac rehabilitation.

Exercise	Regimen
**Breathing exercise (3 min)**	10 reps/set, 3 sets
**Upper limbs exercise/active chest trunk mobilization (9 min)**	
Shoulder flexion (anteroposterior chest wall mobilization)	10 reps/set, 3 sets
Shoulder abduction	10 reps/set, 3 sets
Shoulder flexion to the opposite side (posterolateral chest wall mobilization)	10 reps/set, 3 sets
Lateral chest wall mobilization	10 reps/set, 3 sets
**Lower limb exercise (4.33 min/cycle, total 7 cycles, 30 min)**	
Marching	—^a^
Hip abduction-adduction	—
Mini squat	—
Hip-knee flexion	—
Hip flexion with adduction	—
**Cool down: marching**	5 min
**Upper extremity exercise or active chest and trunk mobilization (9 min)**	
Shoulder flexion (anteroposterior chest wall mobilization)	10 reps/set, 3 sets
Shoulder abduction	10 reps/set, 3 sets
Shoulder flexion to the opposite side (posterolateral chest wall mobilization)	10 reps/set, 3 sets
Lateral chest wall mobilization	10 reps/set, 3 sets
**Breathing exercise (3 min)**	10 reps/set, 3 sets

a Not applicable.

**Figure 1.**
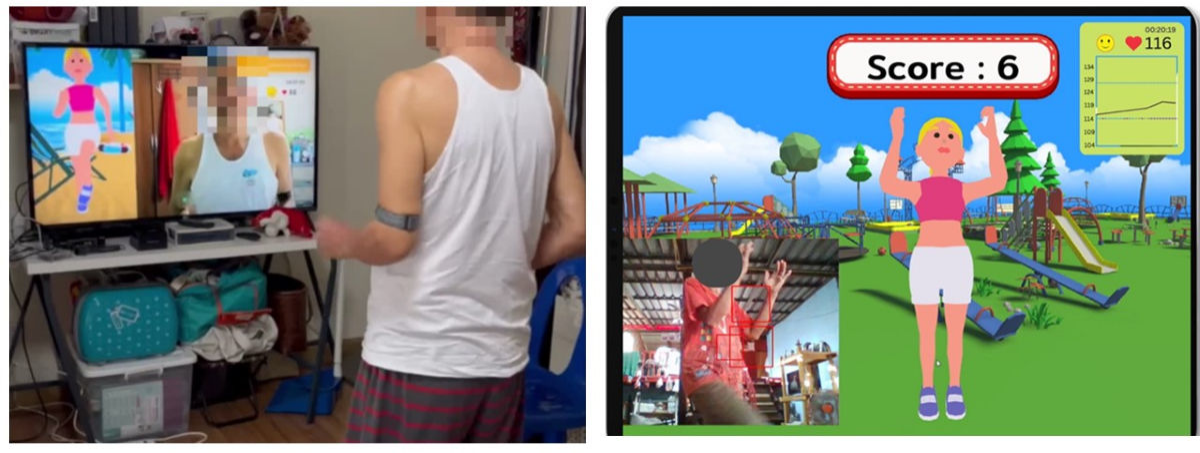
Virtual reality exercise.

We developed a nonimmersive VR exercise in which the user interacts indirectly with a virtual environment through 2D flat-screen programs [17]. In addition, a Polar Verity Sense (optical HR sensor; Polar Electro) device was used to monitor the HR and display it in real time on the screen (Figure 1). The VR exercise program was set at 40%‐59% of the HRR with automatic HR adjustment for individuals. That is, if participants performed lower than 40% HRR, the VR exercise program was sped up until the participant achieved 40%‐59% HRR. In contrast, if the participant performed higher than the target HR (40%‐59% HRR), the program was slowed down until the participant achieved 40%‐59% HRR. The aerobic exercise protocol was reviewed by physical therapists and physical medicine and rehabilitation specialists.

Before and after the 8-week intervention program, all participants underwent respiratory assessment using a respiratory pressure meter (Micro RPM, Micro Medical Ltd). Maximal inspiratory pressure (MIP) and maximal expiratory pressure (MEP) were defined as inspiratory and expiratory muscle strength, respectively. The protocol adhered to the American Thoracic Society’s (ATS’) and European Respiratory Society’s (ERS’) guidelines [18]. Briefly, the participant was requested to exhale until the residual volume was achieved and then to inhale deeply; then, the MIP was recorded. In contrast, the individual inhaled maximally (total lung capacity) and then exhaled quickly; then, MEP was recorded.

A spirometer (MicroLab Mk8 Desktop Spirometer, CareFusion Corp) was used to assess forced vital capacity (FVC) and forced expiratory volume in 1 second (FEV1), following the ATS’ and ERS’ guidelines for respiratory muscle testing [19]. Functional capacity was evaluated using the 6-minute walk test (6MWT) according to the ATS’ statement guidelines for the 6MWT [20]. Briefly, participants were required to walk along a 30-m-long corridor, and the 6MWD was recorded.

An intention-to-treat analysis was conducted, which included all patients randomly assigned to the study group who underwent the intervention program at least once. Two-way mixed ANOVA (2 types×2 times) was used to compare between- and within-group differences in cardiorespiratory performance. Statistical significance was set at *P*<.05. SPSS (version 24; IBM Corp) was used for statistical analyses. In addition, this trial was registered in the Thai Clinical Trial Registry (TCTR20230602001). The study was conducted in line with the CONSORT (Consolidated Standards of Reporting Trials) 2010 statement.

### Ethical Considerations

The study was approved by the Human Research Ethics Committee of Thammasat University (Science; COA number 023/2565) and complied with the tenets of the Declaration of Helsinki, The Belmont Report, Council for International Organizations of Medical Sciences guidelines, and the International Conference on Harmonization’s Good Clinical Practice guidelines. The participants provided informed consent to take part in the study.

## Results

**Figure 2.**
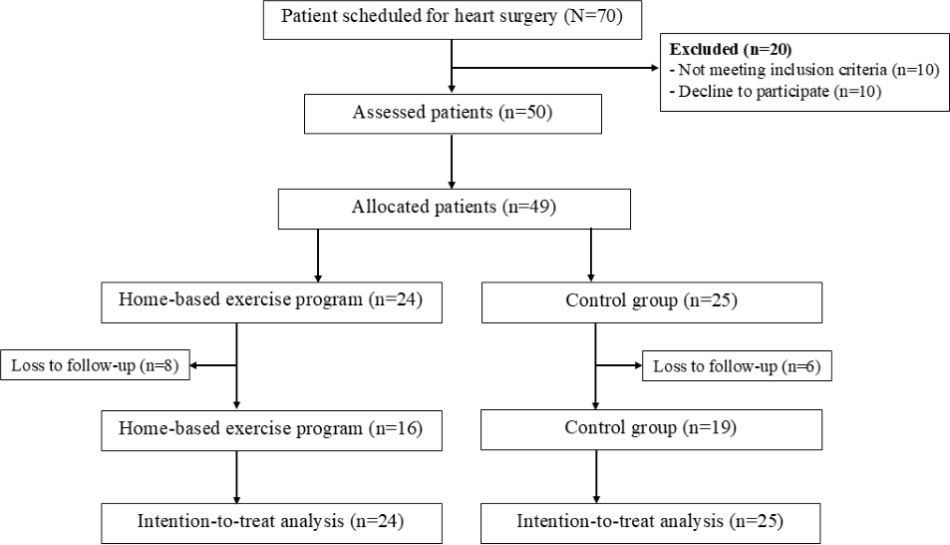
Flow diagram showing the study recruitment process.

Out of 70 undergoing OHS, 20 were excluded because they did not meet the inclusion criteria or were unwilling to participate. Therefore, 50 patients were assessed for eligibility and randomized: 25 participants in the VR group and 25 in the control group. After hospital discharge, for ≥3 weeks, the participants were assessed for functional capacity, respiratory muscle strength, and pulmonary function. However, one participant in the VR group was assessed for respiratory muscle strength and functional capacity but not for pulmonary function because of a technical error. Therefore, data were analyzed for 24 and 25 patients in the VR and control groups, respectively. After completing the 8-week program, 8 participants in the VR and 6 participants in the control group were lost to follow-up. Nevertheless, all 49 participants were included in the intention-to-treat analysis (Figure 2).

The mean age of the participants was 60.36 (SD 11.56) years, and two-thirds of the participants were male (male: n=35, 71.43%; female: n=14, 28.57%). Regarding surgery type, over half of the 49 participants underwent CABG (n=31, 63.27%), followed by valve surgery (n=14, 28.57%), and a combination of CABG and valve surgery (n=4, 8.16%). Dyslipidemia (n=25, 51.02%) was the most common comorbidity, followed by hypertension (n=23, 46.94%) and diabetes mellitus (n=13, 26.53%). There were no significant differences in patients’ characteristics between the VR and control groups (Table 2).

Table 3 presents the cardiopulmonary changes before and after the 8-week intervention program. After the 8-week VR exercise program, there were significant increases in the following outcomes: 6MWD (Δ78.74, SD 15.18 m; *P*<.001), MIP (Δ9.46, SD 2.85 cm H_2_O; *P*=.002), MEP (Δ15.79, SD 4.65 cm H_2_O; *P*=.001), and %FEV1 (Δ4.63, SD 1.61; *P*=.006). In addition, the control group showed an improvement in MIP (Δ9.64, SD 2.78 cm H_2_O; *P*=.001) and %FEV1 (Δ4.04, SD 1.58; *P*=.01) values.

Significant differences in the improvement in 6MWD (Δ78.74, SD 15.18; *P*<.001) were observed between the VR and control groups. However, respiratory muscle strength and pulmonary function were not significantly different between the VR and control groups.

**Table 2.** Characteristics of the participants.

			Total (n=49)	Virtual reality group (n=24)	Control group (n=25)	Statistics^a^	*P* value
Age (years), mean (SD)			60.36 (11.56)	62.75 (7.97)	58.08 (13.97)	*1.429 (47)*	.16
Weight (kg), mean (SD)			63.48 (12.35)	63.55 (11.54)	63.42 (13.32)	*0.038 (47)*	.97
BMI (kg/m^2^), mean (SD)			23.82 (4.42)	24.64 (4.10)	23.05 (4.66)	*1.267 (47)*	.21
**Sex, n (%)**						*3.953 (1)*	.047
	Male		35 (71.43)	14 (40.00)	21 (60.00)		
	Female		14 (28.57)	10 (71.43)	4 (28.57)		
**Types of surgery, n (%)**						4.299 (2)	.12
	CABG^b^		31 (63.27)	16 (51.61)	15 (48.39)		
	Valve surgery		14 (28.57)	8 (57.14)	6 (42.86)		
	CABG + valve surgery		4 (8.16)	0 (0.00)	4 (100.00)		
**Comorbidities, n (%)**							
	**Diabetes mellitus**					0.168 (1)	.68
		History of diabetes mellitus	13 (26.53)	7 (53.85)	6 (46.15)		
		No history of diabetes mellitus	36 (73.47)	17 (47.22)	19 (52.78)		
	**Hypertension**					0.525 (1)	.47
		History of hypertension	23 (46.94)	10 (473.48)	13 (56.52)		
		No history of hypertension	26 (53.06)	14 (53.85)	12 (46.15)		
	**Dyslipidemia**					1.647 (1)	.20
		History of dyslipidemia	25 (51.02)	10 (40.00)	15 (60.00)		
		No history of dyslipidemia	24 (48.98)	14 (58.33)	10 (41.67)		
	**COPD^c^** **or asthma**					1.063 (1)	.30
		History of COPD or asthma	1 (2.04)	1 (100.00)	0 (0.00)		
		No history of COPD or asthma	48 (97.96)	23 (47.92)	25 (52.08)		

a *t* test (*df*) or chi-square (*df*) values (the former are presented in italics).

b CABG: coronary artery bypass graft.

c COPD: chronic obstructive pulmonary disease.

**Table 3.** Cardiorespiratory performance after the 8-week home-based rehabilitation exercise program.

Variable		Virtual reality group	Control group	Mean difference (virtual reality versus control group), mean (SD)	*P* value between groups
**6-minute walk distance (m)**					
	Before, mean (SD)	273.64 (99.10)	270.25 (89.96)	3.39 (27.02)	.90
	After, mean (SD)	352.38 (80.71)	286.08 (98.83)	66.29 (25.84)	.01
	*P* value within groups	<.001	.29	N/A^a^	N/A
**Maximal inspiratory pressure (cm H**_**2**_ **O)**					
	Before, mean (SD)	59.83 (21.39)	58.08 (26.94)	1.75 (6.97)	.80
	After, mean (SD)	69.29 (21.69)	67.72 (34.57)	1.57 (8.29)	.85
	*P* value within groups	.002	.001	N/A	N/A
**Maximal expiratory pressure (cm H**_**2**_ **O)**					
	Before, mean (SD)	57.50 (25.08)	59.04 (24.79)	–1.54 (7.73)	.83
	After, mean (SD)	73.29 (33.60)	63.12 (24.78)	10.17 (8.41)	.23
	*P* value within groups	.001	.38	N/A	N/A
**Forced vital capacity (%)**					
	Before, mean (SD)	60.54 (16.41)	63.12 (17.39)	–2.58 (4.83)	.60
	After, mean (SD)	63.50 (15.10)	63.96 (16.13)	–0.46 (4.47)	.92
	*P* value within groups	.058	.58	N/A	N/A
**Forced expiratory volume for 1 second (%)**					
	Before, mean (SD)	68.04 (18.43)	67.96 (22.08)	0.08 (5.82)	.99
	After, mean (SD)	72.67 (16.40)	72.00 (19.47)	0.67 (5.15)	.90
	*P* value within groups	.006	.01	N/A	N/A

a N/A: not applicable.

## Discussion

### Principal Findings

This study aimed to determine the effects of home-based VR exercise on cardiorespiratory performance in patients who underwent OHS and were in phase II CR. This study demonstrated that a home-based VR exercise program improved functional capacity, %FVC, and respiratory muscle strength (ie, MIP and MEP). In contrast, MIP increased after the 8-week program in the control group. In addition, functional capacity increased in the VR group compared to that in the control group after the 8-week program.

An improved 6MWD (78.74, SD 15.18 m) was noted in the VR group but not in the control group (mean 6MWD 15.83, SD 14.87 m). A systematic review and meta-analysis of 10 randomized controlled trials reported the benefit of VR exercise for 6MWD in participants undergoing CR with a mean 6MWD of 49.55 m (95% CI 30.59‐68.52) [21]. However, the study reported on different phases of CR (eg, phases I, II, and III) and different cardiac diseases (eg, heart failure, ischemic heart disease, and having undergone cardiac surgery in phase I). In addition, the minimal clinically important difference for 6MWT was 36.11 m in 89 inpatients in CR phase I who had undergone elective cardiac surgery (ie, CABG) [22]. In phase II CR, the minimal clinically important difference for the 6MWD was 25 m for patients who underwent an 8-week CR program after acute coronary syndrome [23]. Therefore, the 8-week VR exercise showed benefits in improving functional capacity both clinically and statistically in patients with cardiac conditions who had undergone elective surgery.

Moderate-intensity exercise improves cardiovascular function by increasing HR, cardiac output, blood circulation, endothelial function, capillary density, and nitric oxide bioavailability, which enhance the ability to use oxygen to produce energy for activity [24]. In addition, aerobic exercise causes cardiovascular adaptation by increasing oxygen consumption, which improves cardiorespiratory fitness and endurance capacity [25]. In this study, the VR group showed significant improvements compared to the control group. The VR exercise protocol followed the American College of Sports Medicine guidelines [16] and involved moderate-intensity aerobic exercise (40%‐59% HRR), which might improve cardiorespiratory performance. Several studies have supported the positive effects of VR based on aerobic exercise, which can improve functional capacity [14,26-29]. Therefore, the results of this study are in line with those of previous studies regarding the positive effects of VR exercise on functional capacity and cardiovascular endurance.

After 8 weeks, both the VR and control groups demonstrated significant improvements in respiratory muscle strength and pulmonary function. A previous study found that VR-based aerobic exercise helped to improve respiratory activity, effectively contributing to the evaluation of cardiorespiratory conditioning [12]. Aerobic exercise improves pulmonary ventilation (increases respiratory rate and tidal volume) and diffusion (increases pulmonary blood flow) [30]. However, this study showed no significant differences in respiratory muscle strength and pulmonary function between the 2 groups. This is probably due to improvements in respiratory muscle strength and pulmonary function in both groups, which occurred after discharge (6‐8 weeks), following recovery from incisional pain, atelectasis, pulmonary problems, and postoperative inflammation, leading to an increased ability to perform respiratory muscle tests [31]. Moreover, respiratory muscle strength decreased on postoperative days 3 and 6 compared to that before surgery, and the healing process might have been consolidated within 2‐4 weeks [32]. Rouhi-Boroujeni et al [33] reported that patients scheduled for CABG had a reduction in % FEV1 from 73.4 (SD 0.5) to 64.6 (SD 12.2) 1 week after surgery and then improved to 68.4 (SD 0.2) 6 months post surgery. Therefore, the recovery of respiratory performance (ie, respiratory muscle strength and pulmonary function) in the 12 months following hospital discharge was observed in both the VR and control groups. Thus, the improvement in pulmonary performance and respiratory muscle strength could be due to the postoperative recovery process [33]. In addition, VR focuses on cardiovascular endurance but is not specific to respiratory muscle strength or pulmonary function, possibly leading to the lack of significant differences in pulmonary performance between the groups. Consequently, VR exercise improved cardiovascular endurance rather than pulmonary performance.

This study had some limitations. This study was in favor of men because the rate of CABG is higher in men than women. Further, the proportion of women in the control group was extremely low, which may affect the comparisons. Besides, we did not record characteristics such as ejection fraction, functional class from the New York Heart Association, laboratory data, and physical activity level. Most of the participants in this study were classified as New York Heart Association class I and II or ambulatory patients. Therefore, the results may not be generalized to all patients in phase II CR. In addition, medication usage is not reported, which might affect the intensity of exercise; however, moderate-intensity exercise can be used with measures of HRR or subjective feeling (ie, the rate of perceived exertion).

### Conclusion

A home-based exercise program improved cardiovascular endurance, pulmonary function, and respiratory muscle strength in patients who underwent OHS and were in phase II CR. However, there were no significant differences between the VR and control groups in pulmonary function and respiratory muscle strength. Therefore, the VR exercise program improves cardiovascular endurance and functional capacity in patients who undergo OHS and are in phase II CR.

## Supplementary material

10.2196/68504 Checklist 1 CONSORT (Consolidated Standards of Reporting Trials) checklist.

## Abbreviations

6MWD: 6-minute walk distance
6MWT: 6-minute walk test
ATS: American Thoracic Society
CABG: coronary artery bypass graft
CONSORT: Consolidated Standards of Reporting Trials
CR: cardiac rehabilitation
CVD: cardiovascular disease
ERS: European Respiratory Society
FEV1: forced expiratory volume in 1 second
FVC: forced vital capacity
HR: heart rate
HRR: heart rate reserve
MEP: maximal expiratory pressure
MIP: maximal inspiratory pressure
OHS: open heart surgery
VR: virtual reality
